# Premature Ventricular Complex–induced Full Pre-excitation in Wolff–Parkinson–White Syndrome: A Case Report

**DOI:** 10.19102/icrm.2026.17025

**Published:** 2026-02-15

**Authors:** Ali Saad Al-Shammari, Robert Spalding, Haider Al-Taii

**Affiliations:** 1 Internal Medicine Department, Baghdad Teaching Hospital, Baghdad, Iraq; 2 Department of Cardiology, University of West Virginia, Camden Clark Medical Center, Parkersburg, WV, USA; 3 Cardiac Ablation Solutions, Abbott Laboratories, Houston, TX, USA; 4 Department of Cardiology, Aultman Deuble Heart & Vascular Hospital, Aultman Health System, Canton, OH, USA

**Keywords:** Catheter ablation, pre-excitation, premature ventricular complexes, Wolff–Parkinson–White syndrome

## Abstract

In this report, we present a unique case of a patient with Wolff–Parkinson–White (WPW) syndrome who demonstrated premature ventricular complexes followed by fully pre-excited beats, highlighting an uncommon electrophysiologic phenomenon, and discuss its diagnostic and therapeutic implications.

## Introduction

Wolff–Parkinson–White (WPW) syndrome is a pre-excitation disorder characterized by the presence of an accessory pathway that allows for anomalous conduction between the atria and ventricles, fusing with conduction via the atrioventricular (AV) node.^1^ This can result in a variety of arrhythmias, most commonly AV re-entrant tachycardia, and may predispose patients to more serious outcomes such as atrial fibrillation with rapid ventricular response.^2^ Premature ventricular complexes (PVCs) are ectopic beats originating in the ventricles and are frequently seen in both healthy individuals and those with structural heart disease. Although PVCs and WPW syndrome are each well-described arrhythmic entities, their coexistence and interaction are rarely reported in the literature. Even more unusual is the observation of a PVC immediately followed by a fully pre-excited beat, suggesting a complex interplay between ventricular ectopy and the accessory pathway.^3^

## Case presentation

**Figure 1:**
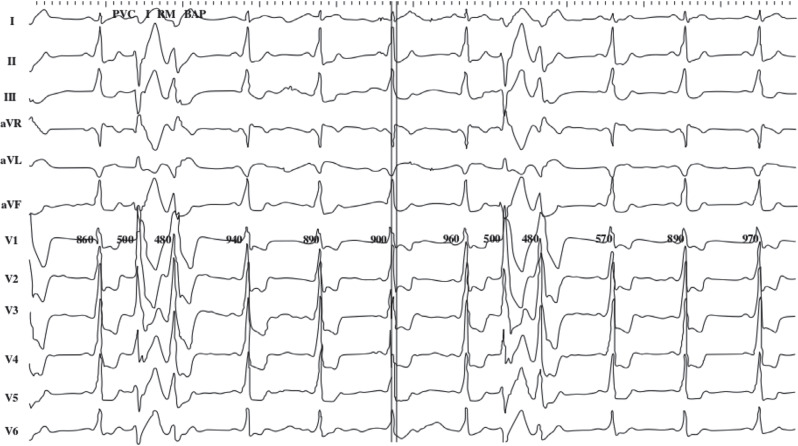
Left-sided delta wave; positive leads V1, II, and III; negative aVL; left anterolateral pathway; and premature ventricular complex with right bundle morphology and superior directed axis.

A 65-year-old man with no prior cardiac history presented to the cardiac electrophysiology clinic with a chief complaint of palpitations and dizziness. A 12-lead electrocardiogram (ECG) showed evidence of pre-excitation and frequent wide complex beats, which were initially thought to represent multifocal PVCs **(Figure 1)**. After a thorough discussion of available options, the patient agreed to proceed with an electrophysiology study and possible catheter ablation.

**Figure 2:**
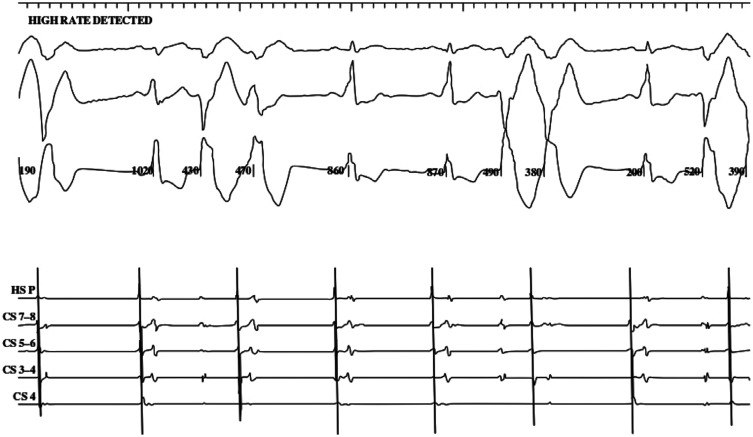
Intracardiac electrograms revealed that the first wide complex beat was a premature ventricular complex, while the second was a fully pre-excited beat.

**Figure 3:**
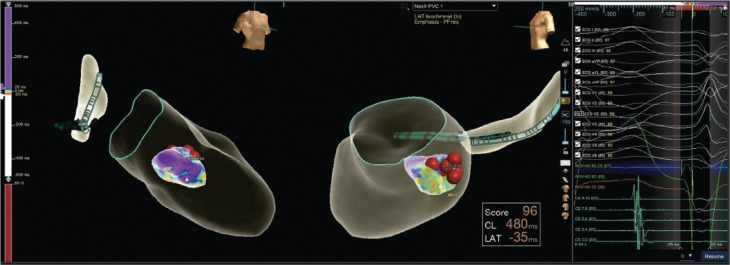
A three-dimensional map of the premature ventricular complex. The red tags show the targeted lesions.

Under moderate sedation, both femoral arterial and venous access were obtained using ultrasound guidance. Three sheaths were placed in the right femoral vein and one sheath was placed in the right femoral artery. Following an intracardiac echocardiography examination, a coronary sinus catheter was placed. Intracardiac electrograms (EGMs) revealed that the first wide complex beat was a PVC, while the second was a fully pre-excited beat **(Figure 2)**. We proceeded with careful three-dimensional (3D) mapping of the PVC via a retrograde aortic approach. The earliest EGM was identified at the posterior medial papillary muscle insertion. Radiofrequency (RF) ablation at this site resulted in the immediate elimination of the PVCs as well as the fully pre-excited beats. **Figure 3** demonstrates red tags on our map pinpointing the targeted ablation sites.

**Figure 4:**
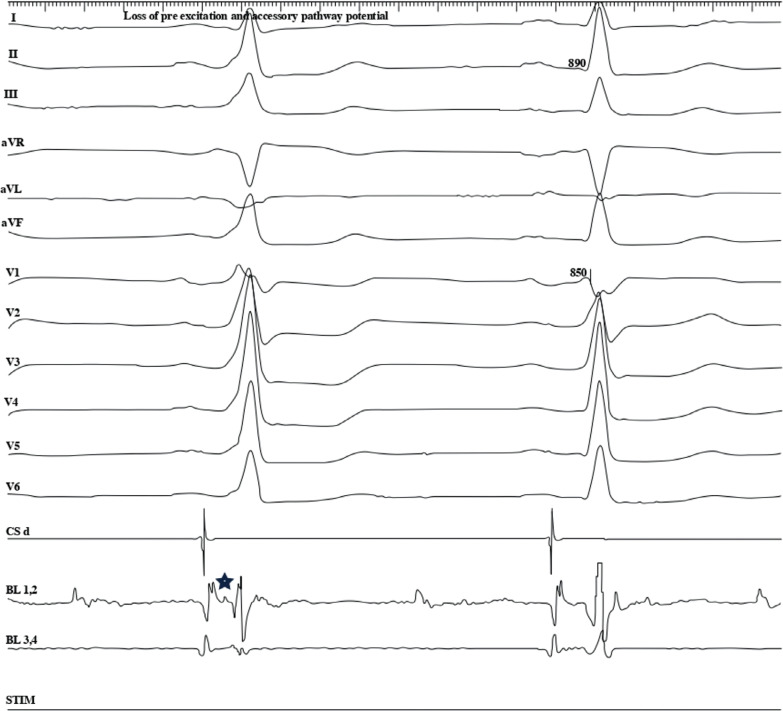
Pathway potential visualized on the first beat with pre-excitation along with the delta wave. After radiofrequency application, loss of pre-excitation can be seen on the second beat.

**Figure 5:**
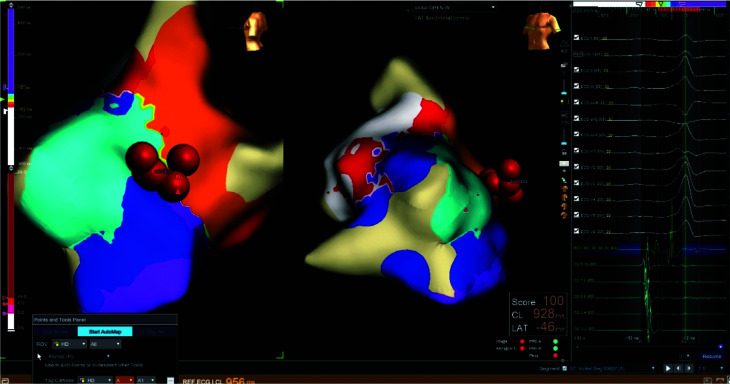
A three-dimensional map of the accessory pathway. The red tags show the targeted lesions.

Attention was then directed to mapping the accessory pathway around the mitral annulus. As mapping progressed around the mitral annulus, the accessory pathway was localized to the left anterolateral region. An accessory pathway potential was identified, and RF application at this site resulted in immediate loss of the pre-excitation pattern **(Figure 4)**. The 3D map vividly captured this area, with red tags marking the precise sites where successful ablation lesions were delivered **(Figure 5)**.

This study was conducted in accordance with the institutional guidelines and approved by the Human Studies Committee (institutional review board) at the College of Medicine, University of Baghdad (reference no. 11443).

## Discussion

This case highlights an uncommon but clinically relevant interaction between PVCs and an accessory pathway in WPW syndrome. While prior reports have relied mainly on surface ECG interpretation, this report is distinctive in that it provides invasive electrophysiologic confirmation of a PVC, followed by a fully pre-excited beat, suggesting retrograde AV nodal penetration resulting in antegrade AV nodal refractoriness, allowing exclusive antegrade conduction via the accessory pathway. This underscores the diagnostic value of recognizing dynamic pre-excitation patterns triggered by ventricular ectopy. A similar mechanism was reported by Longo and Baranchuk, who described PVC-induced concealed retrograde penetration of the AV node or accessory pathway. Their findings, based on surface ECGs, showed variability in post-PVC pre-excitation depending on the degree of antegrade AV nodal delay or whether antegrade block is present.^3^ Our case adds intracardiac evidence and 3D electroanatomic mapping, offering direct visualization of both the PVC origin and accessory pathway.

The clinical impact is significant. Preisendörfer et al. reported inappropriate implantable cardioverter-defibrillator placement in a patient with intermittent pre-excitation due to overlooked conduction clues. In contrast, our case highlights how early recognition of PVC-related conduction changes, supported by invasive mapping, can guide appropriate therapy and prevent unnecessary interventions.^4^

## Conclusion

This case demonstrates the influence of PVCs on antegrade AV nodal and accessory pathway conduction. The PVC rendered the AV node refractory, allowing the subsequent beat to be fully conducted via the accessory pathway, resulting in full pre-excitation. The change in ECG morphology between the PVC and the following pre-excited beat can be explained by the differing origins of the PVC and the location of the accessory pathway.
